# 2-Amino-5-methyl­pyridinium 4-methyl­benzoate

**DOI:** 10.1107/S1600536812050374

**Published:** 2012-12-15

**Authors:** Kaliyaperumal Thanigaimani, Abbas Farhadikoutenaei, Suhana Arshad, Ibrahim Abdul Razak

**Affiliations:** a School of Physics, Universiti Sains Malaysia, 11800 USM, Penang, Malaysia; b Department of Physics, Faculty of Science, University of Mazandaran, Babolsar, Iran

## Abstract

The 4-methyl­benzoate anion of the title salt, C_6_H_9_N_2_^+^·C_8_H_7_O_2_^−^, is nearly planar, with a dihedral angle of 6.26 (10)° between the benzene ring and the carboxyl­ate group. In the crystal, the protonated N atom and the 2-amino group of the cation are hydrogen bonded to the carboxyl­ate O atoms of the anion *via* a pair of N—H⋯O hydrogen bonds with an *R*_2_^2^(8) ring motif, forming an approximately planar ion pair with a dihedral angle of 9.63 (4)° between the pyridinium and benzene rings. The ion pairs are further connected *via* N—H⋯O and weak C—H⋯O hydrogen bonds, forming a two-dimensional network parallel to the *bc* plane.

## Related literature

For background to the chemistry of substituted pyridines, see: Pozharski *et al.* (1997 ▶); Katritzky *et al.* (1996 ▶). For related structures, see: Nahringbauer & Kvick (1977 ▶); Thanigaimani *et al.* (2012*a* ▶,*b* ▶,*c* ▶). For hydrogen-bond motifs, see: Bernstein *et al.* (1995 ▶). For bond-length data, see: Allen *et al.* (1987 ▶). For stability of the temperature controller used for the data collection, see: Cosier & Glazer (1986 ▶).
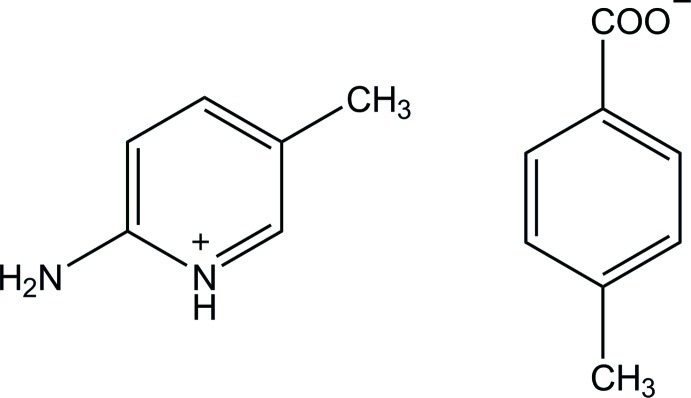


## Experimental

### 

#### Crystal data

C_6_H_9_N_2_^+^·C_8_H_7_O_2_^−^
*M* *_r_* = 244.29
Monoclinic, 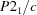

*a* = 9.6315 (5) Å
*b* = 10.8713 (6) Å
*c* = 12.1481 (7) Å
β = 104.093 (1)°
*V* = 1233.71 (12) Å^3^
*Z* = 4
Mo *K*α radiation
μ = 0.09 mm^−1^
*T* = 100 K
0.52 × 0.32 × 0.15 mm

#### Data collection

Bruker SMART APEXII CCD area-detector diffractometer
Absorption correction: multi-scan (*SADABS*; Bruker, 2009 ▶) *T*_min_ = 0.955, *T*_max_ = 0.987
18139 measured reflections
4493 independent reflections
3783 reflections with *I* > 2σ(*I*)
*R*_int_ = 0.027

#### Refinement

*R*[*F* ^2^ > 2σ(*F* ^2^)] = 0.043
*wR*(*F* ^2^) = 0.125
*S* = 1.03
4493 reflections
177 parameters
H atoms treated by a mixture of independent and constrained refinement
Δρ_max_ = 0.43 e Å^−3^
Δρ_min_ = −0.29 e Å^−3^

### 

Data collection: *APEX2* (Bruker, 2009 ▶); cell refinement: *SAINT* (Bruker, 2009 ▶); data reduction: *SAINT*; program(s) used to solve structure: *SHELXTL* (Sheldrick, 2008 ▶); program(s) used to refine structure: *SHELXTL*; molecular graphics: *SHELXTL*; software used to prepare material for publication: *SHELXTL* and *PLATON* (Spek, 2009 ▶).

## Supplementary Material

Crystal structure: contains datablock(s) global, I. DOI: 10.1107/S1600536812050374/is5229sup1.cif

Structure factors: contains datablock(s) I. DOI: 10.1107/S1600536812050374/is5229Isup2.hkl

Supplementary material file. DOI: 10.1107/S1600536812050374/is5229Isup3.cml

Additional supplementary materials:  crystallographic information; 3D view; checkCIF report

## Figures and Tables

**Table 1:** Hydrogen-bond geometry (Å, °)

*D*—H⋯*A*	*D*—H	H⋯*A*	*D*⋯*A*	*D*—H⋯*A*
N2—H1*N*2⋯O2^i^	0.920 (16)	1.832 (16)	2.7469 (11)	172.7 (15)
N2—H2*N*2⋯O1^ii^	0.926 (16)	1.919 (17)	2.8424 (11)	175.4 (15)
N1—H1*N*1⋯O1^i^	0.976 (18)	1.751 (18)	2.7224 (10)	173.1 (16)
C10—H10*A*⋯O2^iii^	0.95	2.34	3.1120 (11)	138

Symmetry codes: (i) 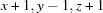; (ii) 
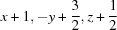
; (iii) 
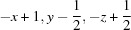
.

**Fig. 1.**
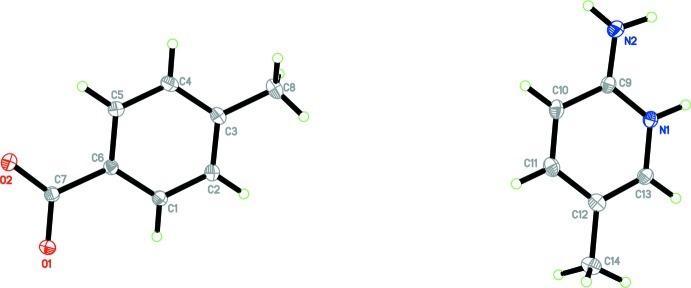
The molecular structure of the title compound with atom labels with 50% probability displacement ellipsoids.

**Fig. 2.**
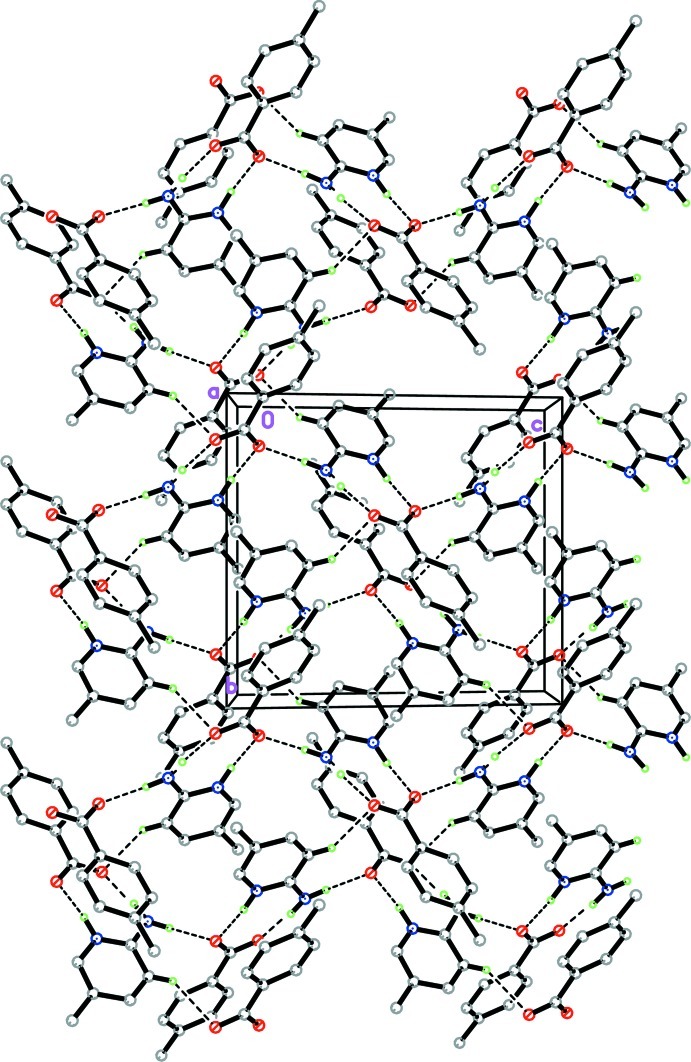
A crystal packing diagram of the title compound. H atoms not involved in the intermolecular interactions (dashed lines) have been omitted for clarity.

## supplementary crystallographic information

### Comment

Pyridine and its derivatives play an important role in heterocyclic chemistry (Pozharski *et al.*, 1997; Katritzky *et al.*, 1996). They are often involved in hydrogen-bond interactions. Related crystal structures of 2-amino-5-methylpyridine (Nahringbauer & Kvick, 1977), 2-amino-5-methylpyridinium 6-oxo-1,6-dihydropyridine-2-carboxylate (Thanigaimani *et al.*, 2012*a*), 2-amino-5-methylpyridinium 3-chlorobenzoate (Thanigaimani *et al.*, 2012*b*) and 2-amino-5-methylpyridinium 2-aminobenzoate (Thanigaimani *et al.*, 2012*c*) have been reported. In order to study potential hydrogen bonding interactions, the crystal structure determination of the title compound (I) was carried out.

The asymmetric unit (Fig. 1) contains one 2-amino-5-methylpyridinium cation and one 4-methylbenzoate anion. In the 2-amino-5-methylpyridinium cation, a wider than normal angle [C9—N1—C13 = 122.15 (8)°] is subtended at the protonated N1 atom. The 2-amino-5-methylpyridinium cation is planar with a maximum deviation of 0.005 (1) Å for atom C9. The dihedral angle between the pyridine (N1/C9–C13) and benzene (C1–C6) rings is 9.63 (4)°. The bond lengths (Allen *et al.*, 1987) and angles are normal. In the crystal packing (Fig. 2), the protonated N1 atom and a nitrogen atom of the 2-amino group (N2) are hydrogen-bonded to the carboxylate oxygen atoms (O1 and O2) *via* a pair of intermolecular N1—H1N1···O1^i^ and N2—H1N2···O2^i^ hydrogen bonds (symmetry code in Table 1), forming a ring motif *R*_2_^2^(8) (Bernstein *et al.*, 1995). Furthermore, these motifs are connected *via* N2—H2N2···O1^ii^ and C10—H10A···O2^iii^ hydrogen bonds (symmetry codes in Table 1), to form a two-dimensional network parallel to the *bc* plane.

### Experimental

Hot methanol solutions (20 ml) of 2-amino5-methylpyridine (54 mg, Aldrich) and 4-methylbenzoic acid (34 mg, Merck) were mixed and warmed over a heating magnetic stirrer hotplate for a few minutes. The resulting solution was allowed to cool slowly at room temperature and crystals of the title compound (I) appeared after a few days.

### Refinement

N-bound H Atoms were located in a difference Fourier maps and refined freely [refined N—H distances 0.976 (18), 0.920 (16) and 0.926 (16) Å]. The remaining hydrogen atoms were positioned geometrically (C—H = 0.95 or 0.98 Å) and were refined using a riding model, with *U*_iso_(H) = 1.2*U*_eq_(C) or 1.5*U*_eq_(methyl C). A rotating group model was used for the methyl group.

### Crystal data

**Table:**

C_6_H_9_N_2_^+^·C_8_H_7_O_2_^−^	*F*(000) = 520
*M**_r_* = 244.29	*D*_x_ = 1.315 Mg m^−^^3^
Monoclinic, *P*2_1_/*c*	Mo *K*α radiation, λ = 0.71073 Å
Hall symbol: -P 2ybc	Cell parameters from 6272 reflections
*a* = 9.6315 (5) Å	θ = 2.6–32.6°
*b* = 10.8713 (6) Å	µ = 0.09 mm^−^^1^
*c* = 12.1481 (7) Å	*T* = 100 K
β = 104.093 (1)°	Block, colourless
*V* = 1233.71 (12) Å^3^	0.52 × 0.32 × 0.15 mm
*Z* = 4	

### Data collection

**Table:**

Bruker SMART APEXII CCD area-detector diffractometer	4493 independent reflections
Radiation source: fine-focus sealed tube	3783 reflections with *I* > 2σ(*I*)
Graphite monochromator	*R*_int_ = 0.027
φ and ω scans	θ_max_ = 32.7°, θ_min_ = 2.6°
Absorption correction: multi-scan (*SADABS*; Bruker, 2009)	*h* = −14→14
*T*_min_ = 0.955, *T*_max_ = 0.987	*k* = −16→16
18139 measured reflections	*l* = −18→18

### Refinement

**Table:**

Refinement on *F*^2^	Primary atom site location: structure-invariant direct methods
Least-squares matrix: full	Secondary atom site location: difference Fourier map
*R*[*F*^2^ > 2σ(*F*^2^)] = 0.043	Hydrogen site location: inferred from neighbouring sites
*wR*(*F*^2^) = 0.125	H atoms treated by a mixture of independent and constrained refinement
*S* = 1.03	*w* = 1/[σ^2^(*F*_o_^2^) + (0.0678*P*)^2^ + 0.3153*P*] where *P* = (*F*_o_^2^ + 2*F*_c_^2^)/3
4493 reflections	(Δ/σ)_max_ = 0.001
177 parameters	Δρ_max_ = 0.43 e Å^−^^3^
0 restraints	Δρ_min_ = −0.29 e Å^−^^3^

### Special details

**Table:**

Experimental. The crystal was placed in the cold stream of an Oxford Cryosystems Cobra open-flow nitrogen cryostat (Cosier & Glazer, 1986) operating at 100.0 (1) K.
Geometry. All e.s.d.'s (except the e.s.d. in the dihedral angle between two l.s. planes) are estimated using the full covariance matrix. The cell e.s.d.'s are taken into account individually in the estimation of e.s.d.'s in distances, angles and torsion angles; correlations between e.s.d.'s in cell parameters are only used when they are defined by crystal symmetry. An approximate (isotropic) treatment of cell e.s.d.'s is used for estimating e.s.d.'s involving l.s. planes.
Refinement. Refinement of *F*^2^ against ALL reflections. The weighted *R*-factor *wR* and goodness of fit *S* are based on *F*^2^, conventional *R*-factors *R* are based on *F*, with *F* set to zero for negative *F*^2^. The threshold expression of *F*^2^ > σ(*F*^2^) is used only for calculating *R*-factors(gt) *etc*. and is not relevant to the choice of reflections for refinement. *R*-factors based on *F*^2^ are statistically about twice as large as those based on *F*, and *R*- factors based on ALL data will be even larger.

### Fractional atomic coordinates and isotropic or equivalent isotropic displacement parameters (Å^2^)

**Table:**

	*x*	*y*	*z*	*U*_iso_*/*U*_eq_	
O1	0.07967 (7)	1.13827 (6)	0.07082 (5)	0.01774 (14)	
O2	0.20480 (8)	1.12140 (6)	−0.06122 (6)	0.01914 (15)	
C1	0.23323 (9)	0.93283 (8)	0.19086 (7)	0.01486 (16)	
H1A	0.1613	0.9680	0.2224	0.018*	
C2	0.31890 (10)	0.83760 (8)	0.24780 (7)	0.01618 (16)	
H2A	0.3048	0.8090	0.3182	0.019*	
C3	0.42490 (9)	0.78366 (8)	0.20317 (7)	0.01589 (16)	
C4	0.44248 (10)	0.82614 (9)	0.09878 (8)	0.01896 (18)	
H4A	0.5132	0.7900	0.0665	0.023*	
C5	0.35734 (10)	0.92094 (9)	0.04167 (7)	0.01693 (17)	
H5A	0.3702	0.9484	−0.0294	0.020*	
C6	0.25310 (9)	0.97626 (8)	0.08768 (7)	0.01277 (15)	
C7	0.17236 (9)	1.08592 (8)	0.02773 (7)	0.01374 (15)	
C8	0.51917 (10)	0.68238 (9)	0.26587 (8)	0.02109 (19)	
H8A	0.5215	0.6869	0.3469	0.032*	
H8B	0.6164	0.6921	0.2556	0.032*	
H8C	0.4808	0.6024	0.2358	0.032*	
N1	0.94348 (8)	0.32387 (7)	0.93651 (6)	0.01462 (15)	
N2	1.04273 (9)	0.28128 (8)	0.78450 (7)	0.01928 (16)	
C9	0.95687 (9)	0.35009 (8)	0.83058 (7)	0.01471 (16)	
C10	0.87654 (10)	0.45002 (9)	0.77234 (7)	0.01714 (17)	
H10A	0.8825	0.4703	0.6976	0.021*	
C11	0.79056 (10)	0.51697 (8)	0.82424 (8)	0.01771 (17)	
H11A	0.7373	0.5839	0.7847	0.021*	
C12	0.77883 (9)	0.48901 (8)	0.93576 (8)	0.01643 (16)	
C13	0.85738 (9)	0.39091 (8)	0.98802 (7)	0.01558 (16)	
H13A	0.8517	0.3689	1.0625	0.019*	
C14	0.68490 (11)	0.56433 (9)	0.99240 (9)	0.02191 (19)	
H14A	0.6859	0.5287	1.0667	0.033*	
H14B	0.5867	0.5644	0.9450	0.033*	
H14C	0.7209	0.6489	1.0024	0.033*	
H1N2	1.0977 (16)	0.2237 (15)	0.8310 (13)	0.035 (4)*	
H2N2	1.0599 (17)	0.3085 (15)	0.7168 (14)	0.035 (4)*	
H1N1	0.9942 (18)	0.2547 (17)	0.9795 (15)	0.046 (5)*	

### Atomic displacement parameters (Å^2^)

**Table:**

	*U*^11^	*U*^22^	*U*^33^	*U*^12^	*U*^13^	*U*^23^
O1	0.0208 (3)	0.0182 (3)	0.0149 (3)	0.0068 (2)	0.0055 (2)	0.0002 (2)
O2	0.0245 (3)	0.0186 (3)	0.0158 (3)	0.0042 (2)	0.0075 (2)	0.0036 (2)
C1	0.0150 (4)	0.0143 (4)	0.0157 (4)	0.0013 (3)	0.0046 (3)	0.0008 (3)
C2	0.0175 (4)	0.0155 (4)	0.0156 (4)	0.0011 (3)	0.0041 (3)	0.0021 (3)
C3	0.0149 (4)	0.0135 (4)	0.0182 (4)	0.0011 (3)	0.0021 (3)	0.0004 (3)
C4	0.0179 (4)	0.0184 (4)	0.0221 (4)	0.0052 (3)	0.0076 (3)	0.0013 (3)
C5	0.0184 (4)	0.0174 (4)	0.0162 (4)	0.0030 (3)	0.0064 (3)	0.0015 (3)
C6	0.0129 (3)	0.0119 (3)	0.0131 (3)	0.0002 (3)	0.0023 (3)	−0.0007 (3)
C7	0.0157 (4)	0.0127 (3)	0.0122 (3)	0.0002 (3)	0.0021 (3)	−0.0012 (3)
C8	0.0197 (4)	0.0171 (4)	0.0249 (4)	0.0044 (3)	0.0024 (3)	0.0035 (3)
N1	0.0161 (3)	0.0144 (3)	0.0134 (3)	0.0003 (2)	0.0037 (2)	0.0022 (2)
N2	0.0229 (4)	0.0206 (4)	0.0159 (3)	0.0040 (3)	0.0076 (3)	0.0036 (3)
C9	0.0158 (4)	0.0146 (4)	0.0135 (3)	−0.0019 (3)	0.0032 (3)	0.0012 (3)
C10	0.0187 (4)	0.0170 (4)	0.0151 (4)	0.0001 (3)	0.0028 (3)	0.0044 (3)
C11	0.0172 (4)	0.0149 (4)	0.0203 (4)	0.0003 (3)	0.0032 (3)	0.0036 (3)
C12	0.0149 (4)	0.0144 (4)	0.0198 (4)	−0.0014 (3)	0.0041 (3)	0.0001 (3)
C13	0.0165 (4)	0.0160 (4)	0.0146 (4)	−0.0012 (3)	0.0043 (3)	0.0003 (3)
C14	0.0203 (4)	0.0207 (4)	0.0259 (4)	0.0030 (3)	0.0077 (3)	−0.0002 (3)

### Geometric parameters (Å, º)

**Table:**

O1—C7	1.2737 (10)	N1—C9	1.3545 (11)
O2—C7	1.2564 (11)	N1—C13	1.3649 (11)
C1—C6	1.3956 (12)	N1—H1N1	0.976 (18)
C1—C2	1.3975 (12)	N2—C9	1.3351 (12)
C1—H1A	0.9500	N2—H1N2	0.920 (16)
C2—C3	1.3962 (13)	N2—H2N2	0.926 (16)
C2—H2A	0.9500	C9—C10	1.4187 (12)
C3—C4	1.3981 (13)	C10—C11	1.3665 (13)
C3—C8	1.5100 (12)	C10—H10A	0.9500
C4—C5	1.3924 (12)	C11—C12	1.4195 (13)
C4—H4A	0.9500	C11—H11A	0.9500
C5—C6	1.3984 (12)	C12—C13	1.3708 (12)
C5—H5A	0.9500	C12—C14	1.5052 (13)
C6—C7	1.5101 (12)	C13—H13A	0.9500
C8—H8A	0.9800	C14—H14A	0.9800
C8—H8B	0.9800	C14—H14B	0.9800
C8—H8C	0.9800	C14—H14C	0.9800
			
C6—C1—C2	120.10 (8)	C9—N1—C13	122.15 (8)
C6—C1—H1A	120.0	C9—N1—H1N1	121.3 (10)
C2—C1—H1A	120.0	C13—N1—H1N1	116.6 (10)
C3—C2—C1	121.23 (8)	C9—N2—H1N2	116.5 (10)
C3—C2—H2A	119.4	C9—N2—H2N2	117.1 (10)
C1—C2—H2A	119.4	H1N2—N2—H2N2	124.3 (14)
C2—C3—C4	118.36 (8)	N2—C9—N1	119.50 (8)
C2—C3—C8	121.14 (8)	N2—C9—C10	122.56 (8)
C4—C3—C8	120.51 (8)	N1—C9—C10	117.94 (8)
C5—C4—C3	120.64 (8)	C11—C10—C9	119.73 (8)
C5—C4—H4A	119.7	C11—C10—H10A	120.1
C3—C4—H4A	119.7	C9—C10—H10A	120.1
C4—C5—C6	120.81 (8)	C10—C11—C12	121.62 (8)
C4—C5—H5A	119.6	C10—C11—H11A	119.2
C6—C5—H5A	119.6	C12—C11—H11A	119.2
C1—C6—C5	118.84 (8)	C13—C12—C11	116.45 (8)
C1—C6—C7	122.21 (7)	C13—C12—C14	122.48 (8)
C5—C6—C7	118.84 (7)	C11—C12—C14	121.08 (8)
O2—C7—O1	124.21 (8)	N1—C13—C12	122.11 (8)
O2—C7—C6	116.77 (8)	N1—C13—H13A	118.9
O1—C7—C6	118.99 (7)	C12—C13—H13A	118.9
C3—C8—H8A	109.5	C12—C14—H14A	109.5
C3—C8—H8B	109.5	C12—C14—H14B	109.5
H8A—C8—H8B	109.5	H14A—C14—H14B	109.5
C3—C8—H8C	109.5	C12—C14—H14C	109.5
H8A—C8—H8C	109.5	H14A—C14—H14C	109.5
H8B—C8—H8C	109.5	H14B—C14—H14C	109.5
			
C6—C1—C2—C3	−0.32 (13)	C1—C6—C7—O1	−2.27 (12)
C1—C2—C3—C4	−0.82 (13)	C5—C6—C7—O1	−178.40 (8)
C1—C2—C3—C8	178.86 (8)	C13—N1—C9—N2	179.99 (8)
C2—C3—C4—C5	0.77 (14)	C13—N1—C9—C10	−0.73 (13)
C8—C3—C4—C5	−178.91 (9)	N2—C9—C10—C11	−179.98 (9)
C3—C4—C5—C6	0.41 (14)	N1—C9—C10—C11	0.77 (13)
C2—C1—C6—C5	1.49 (13)	C9—C10—C11—C12	−0.17 (14)
C2—C1—C6—C7	−174.64 (8)	C10—C11—C12—C13	−0.48 (13)
C4—C5—C6—C1	−1.54 (13)	C10—C11—C12—C14	179.36 (9)
C4—C5—C6—C7	174.72 (8)	C9—N1—C13—C12	0.07 (13)
C1—C6—C7—O2	175.94 (8)	C11—C12—C13—N1	0.54 (13)
C5—C6—C7—O2	−0.19 (12)	C14—C12—C13—N1	−179.30 (8)

### Hydrogen-bond geometry (Å, º)

**Table:**

*D*—H···*A*	*D*—H	H···*A*	*D*···*A*	*D*—H···*A*
N2—H1*N*2···O2^i^	0.920 (16)	1.832 (16)	2.7469 (11)	172.7 (15)
N2—H2*N*2···O1^ii^	0.926 (16)	1.919 (17)	2.8424 (11)	175.4 (15)
N1—H1*N*1···O1^i^	0.976 (18)	1.751 (18)	2.7224 (10)	173.1 (16)
C10—H10*A*···O2^iii^	0.95	2.34	3.1120 (11)	138

Symmetry codes: (i) *x*+1, *y*−1, *z*+1; (ii) *x*+1, −*y*+3/2, *z*+1/2; (iii) −*x*+1, *y*−1/2, −*z*+1/2.
